# Diethyl 4-acetyl-5-(2-nitro­phen­yl)pyrrolidine-2,2-dicarboxyl­ate

**DOI:** 10.1107/S1600536810045691

**Published:** 2010-11-17

**Authors:** Long He

**Affiliations:** a College of Chemistry and Chemical Engineering, China West Normal University, Nanchong 637002, People’s Republic of China

## Abstract

The title compound, C_18_H_22_N_2_O_7_, was synthesized by the 1,3-dipolar cyclo­addition reaction of but-3-en-2-one, diethyl 2-amino­malonate and 2-nitro­benzaldehyde. In the mol­ecule, the pyrrolidine ring possesses an envelope conformation. Inter­molecular N—H⋯O and C—H⋯O hydrogen bonds are present in the crystal structure.

## Related literature

For the biological activity of pyrrolidine derivatives, see: Coldham & Hufton (2005 ▶); Grigg (1995 ▶); Kravchenko *et al.* (2005 ▶); Nair & Suja (2007 ▶); Pandey *et al.* (2006 ▶); Sardina & Rapoport (1996 ▶); Witherup *et al.* (1995 ▶). For a related structure, see: He (2009 ▶).
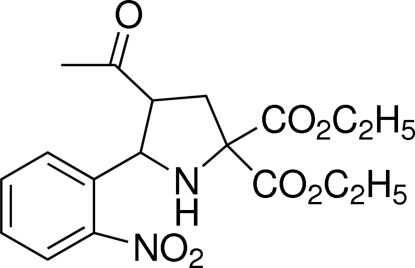


## Experimental

### 

#### Crystal data

C_18_H_22_N_2_O_7_
*M* *_r_* = 378.38
Monoclinic, 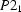

*a* = 10.687 (5) Å
*b* = 7.760 (5) Å
*c* = 12.030 (5) Å
β = 97.455 (5)°
*V* = 989.2 (9) Å^3^
*Z* = 2
Cu *K*α radiation
μ = 0.83 mm^−1^
*T* = 291 K
0.38 × 0.36 × 0.30 mm

#### Data collection

Oxford Diffraction Gemini S Ultra diffractometer
Absorption correction: multi-scan (*CrysAlis PRO*; Oxford Diffraction, 2009 ▶) *T*_min_ = 0.743, *T*_max_ = 0.789
19238 measured reflections
2105 independent reflections
1915 reflections with *I* > 2σ(*I*)
*R*_int_ = 0.023

#### Refinement

*R*[*F* ^2^ > 2σ(*F* ^2^)] = 0.081
*wR*(*F* ^2^) = 0.166
*S* = 1.04
2105 reflections
249 parameters
42 restraints
H atoms treated by a mixture of independent and constrained refinement
Δρ_max_ = 0.33 e Å^−3^
Δρ_min_ = −0.42 e Å^−3^

### 

Data collection: *CrysAlis CCD* (Oxford Diffraction, 2008 ▶); cell refinement: *CrysAlis RED* (Oxford Diffraction, 2008 ▶); data reduction: *CrysAlis RED*; program(s) used to solve structure: *SHELXS97* (Sheldrick, 2008 ▶); program(s) used to refine structure: *SHELXL97* (Sheldrick, 2008 ▶); molecular graphics: *ORTEP-3* (Farrugia, 1997 ▶); software used to prepare material for publication: *SHELXL97*.

## Supplementary Material

Crystal structure: contains datablocks global, I. DOI: 10.1107/S1600536810045691/xu5078sup1.cif

Structure factors: contains datablocks I. DOI: 10.1107/S1600536810045691/xu5078Isup2.hkl

Additional supplementary materials:  crystallographic information; 3D view; checkCIF report

## Figures and Tables

**Table 1:** Hydrogen-bond geometry (Å, °)

*D*—H⋯*A*	*D*—H	H⋯*A*	*D*⋯*A*	*D*—H⋯*A*
N2—H10⋯O2^i^	0.92 (5)	2.51 (6)	3.244 (9)	138 (5)
C18—H18*A*⋯O4^ii^	0.96	2.53	3.460 (15)	162 (6)

Symmetry codes: (i) 
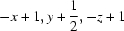
; (ii) 
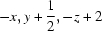
.

**Fig. 1.**
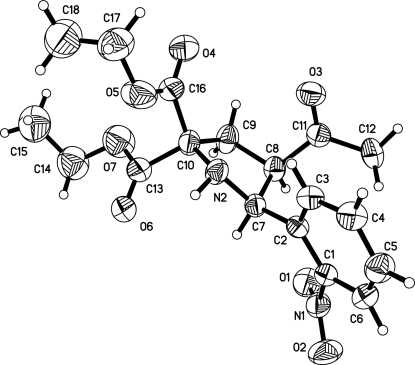
The molecular structure of (I) with 30% probability displacement ellipsoids (arbitrary spheres for H atoms).

## supplementary crystallographic information

### Comment

Substituted pyrrolidine compound is an important class of heterocyclic compounds with wide spread applications to the synthesis of biologically active compounds and natural products (Coldham *et al.*, 2005; Grigg, 1995; Kravchenko *et al.*, 2005; Nair *et al.*, 2007; Pandey *et al.*, 2006; Sardina *et al.*, 1996; Witherup *et al.* 1995). Its crystal structure is reported here.

The molecular structure of (I) is shown in Fig. 1. Bond lengths and angles in (I) are normal. The pyrrolidine ring possesses an envelope conformation. The crystal packing is stabilized by N—H···0 and C—H···0 hydrogen bonding (Table 1).

### Experimental

2-Nitrobenzaldehyde (0.018 g, 0.12 mmol) and diethyl 2-aminomalonate (0.017 g, 0.1 mmol) were added to a solution of methyl but-3-en-2-one (0.014 g, 0.2 mmol) in dichloromethane (2 ml). To the stirred mixture, phosphenous acid (5 mg, 0.01 mmol) was added. After the mixture had been stirred at 293 K for 48 h, the reaction was quenched with a saturated solution of sodium bicarbonate (5 ml). The mixture was extracted with ethyl acetate, evaporated and separated by flash chromatograghy. A colourless powder was obtained. Single crystals suitable for X-ray diffraction were obtained by slow evaporation of an ethyl acetate solution.

### Refinement

H atom on N atom was located in a difference Fourier map and refined isotropically. The carbon-bound hydrogen atoms were placed in calculated positions, with C—H = 0.93–0.98 Å, and refined using a riding model, with *U*_iso_(H) = 1.5*U*_eq_(C) for methyl H atoms and *U*_iso_(H) = 1.2*U*_eq_(C) for the others.

### Crystal data

**Table:**

C_18_H_22_N_2_O_7_	*F*(000) = 400
*M**_r_* = 378.38	*D*_x_ = 1.270 Mg m^−^^3^
Monoclinic, *P*2_1_	Cu *K*α radiation, λ = 1.54184 Å
Hall symbol: P 2yb	Cell parameters from 11509 reflections
*a* = 10.687 (5) Å	θ = 4.2–72.3°
*b* = 7.760 (5) Å	µ = 0.83 mm^−^^1^
*c* = 12.030 (5) Å	*T* = 291 K
β = 97.455 (5)°	Block, colorless
*V* = 989.2 (9) Å^3^	0.38 × 0.36 × 0.30 mm
*Z* = 2	

### Data collection

**Table:**

Oxford Diffraction Gemini S Ultra diffractometer	2105 independent reflections
Radiation source: Enhance Ultra (Cu) X-ray Source	1915 reflections with *I* > 2σ(*I*)
mirror	*R*_int_ = 0.023
ω scans	θ_max_ = 72.6°, θ_min_ = 4.2°
Absorption correction: multi-scan (*CrysAlis PRO*; Oxford Diffraction, 2009)	*h* = −13→13
*T*_min_ = 0.743, *T*_max_ = 0.789	*k* = −8→9
19238 measured reflections	*l* = −14→14

### Refinement

**Table:**

Refinement on *F*^2^	Secondary atom site location: difference Fourier map
Least-squares matrix: full	Hydrogen site location: inferred from neighbouring sites
*R*[*F*^2^ > 2σ(*F*^2^)] = 0.081	H atoms treated by a mixture of independent and constrained refinement
*wR*(*F*^2^) = 0.166	*w* = 1/[σ^2^(*F*_o_^2^) + (0.025*P*)^2^ + 1.3702*P*] where *P* = (*F*_o_^2^ + 2*F*_c_^2^)/3
*S* = 1.04	(Δ/σ)_max_ < 0.001
2105 reflections	Δρ_max_ = 0.33 e Å^−^^3^
249 parameters	Δρ_min_ = −0.42 e Å^−^^3^
42 restraints	Extinction correction: *SHELXL97* (Sheldrick, 2008), Fc^*^=kFc[1+0.001xFc^2^λ^3^/sin(2θ)]^-1/4^
Primary atom site location: structure-invariant direct methods	Extinction coefficient: 0.017 (3)

### Special details

**Table:**

Geometry. All e.s.d.'s (except the e.s.d. in the dihedral angle between two l.s. planes) are estimated using the full covariance matrix. The cell e.s.d.'s are taken into account individually in the estimation of e.s.d.'s in distances, angles and torsion angles; correlations between e.s.d.'s in cell parameters are only used when they are defined by crystal symmetry. An approximate (isotropic) treatment of cell e.s.d.'s is used for estimating e.s.d.'s involving l.s. planes.
Refinement. Refinement of *F*^2^ against ALL reflections. The weighted *R*-factor *wR* and goodness of fit *S* are based on *F*^2^, conventional *R*-factors *R* are based on *F*, with *F* set to zero for negative *F*^2^. The threshold expression of *F*^2^ > σ(*F*^2^) is used only for calculating *R*-factors(gt) *etc*. and is not relevant to the choice of reflections for refinement. *R*-factors based on *F*^2^ are statistically about twice as large as those based on *F*, and *R*- factors based on ALL data will be even larger.

### Fractional atomic coordinates and isotropic or equivalent isotropic displacement parameters (Å^2^)

**Table:**

	*x*	*y*	*z*	*U*_iso_*/*U*_eq_	
O3	0.0038 (5)	0.2421 (8)	0.5230 (4)	0.0881 (16)	
N2	0.2698 (5)	0.3189 (8)	0.6658 (4)	0.0681 (15)	
O5	0.1828 (5)	0.4223 (10)	0.8609 (6)	0.117 (2)	
C3	0.2453 (6)	0.4941 (9)	0.4653 (6)	0.0641 (16)	
H3	0.1941	0.5184	0.5202	0.077*	
O1	0.4209 (6)	0.0223 (8)	0.4138 (5)	0.0957 (18)	
C2	0.3126 (5)	0.3415 (9)	0.4718 (5)	0.0566 (14)	
O6	0.4399 (5)	0.1838 (10)	0.8411 (4)	0.108 (2)	
O4	0.0249 (5)	0.2457 (11)	0.8118 (5)	0.112 (2)	
O2	0.5571 (5)	0.1618 (10)	0.3371 (5)	0.120 (2)	
C8	0.1956 (6)	0.0846 (9)	0.5533 (5)	0.0631 (16)	
H8	0.2247	−0.0192	0.5180	0.076*	
N1	0.4602 (6)	0.1556 (10)	0.3797 (5)	0.0784 (17)	
C7	0.3030 (5)	0.2205 (9)	0.5697 (4)	0.0589 (15)	
H7	0.3841	0.1624	0.5905	0.071*	
C1	0.3875 (5)	0.3157 (10)	0.3870 (5)	0.0605 (15)	
C16	0.1311 (7)	0.2862 (12)	0.8096 (6)	0.079 (2)	
C4	0.2514 (6)	0.6116 (10)	0.3802 (6)	0.0776 (19)	
H4	0.2028	0.7113	0.3771	0.093*	
C12	0.0663 (7)	0.1228 (11)	0.3583 (5)	0.085 (2)	
H12A	−0.0097	0.1760	0.3228	0.128*	
H12B	0.0620	0.0007	0.3455	0.128*	
H12C	0.1375	0.1694	0.3274	0.128*	
C11	0.0803 (6)	0.1578 (9)	0.4820 (5)	0.0649 (16)	
O7	0.2881 (6)	0.0324 (11)	0.9076 (6)	0.130 (2)	
C10	0.2269 (6)	0.1943 (11)	0.7453 (5)	0.0721 (19)	
C6	0.3975 (6)	0.4353 (11)	0.3036 (6)	0.0732 (19)	
H6	0.4511	0.4149	0.2500	0.088*	
C13	0.3357 (7)	0.1387 (14)	0.8371 (6)	0.092 (2)	
C9	0.1723 (8)	0.0442 (10)	0.6740 (5)	0.080 (2)	
H9A	0.0826	0.0330	0.6782	0.096*	
H9B	0.2137	−0.0625	0.6995	0.096*	
C5	0.3294 (7)	0.5809 (11)	0.3001 (6)	0.084 (2)	
H5	0.3350	0.6611	0.2435	0.101*	
C17	0.1066 (10)	0.5318 (17)	0.9259 (8)	0.128 (3)	
H17A	0.0174	0.5231	0.8985	0.154*	
H17B	0.1325	0.6515	0.9246	0.154*	
C14	0.3850 (10)	−0.0385 (18)	1.0007 (8)	0.137 (3)	
H14A	0.3874	−0.1634	0.9994	0.165*	
H14B	0.4687	0.0064	0.9954	0.165*	
C15	0.3341 (11)	0.0277 (19)	1.1036 (8)	0.153 (3)	
H15A	0.3856	−0.0141	1.1694	0.229*	
H15B	0.2490	−0.0121	1.1036	0.229*	
H15C	0.3352	0.1514	1.1034	0.229*	
C18	0.1366 (11)	0.455 (2)	1.0415 (9)	0.158 (3)	
H18A	0.0896	0.5141	1.0925	0.237*	
H18B	0.2253	0.4664	1.0663	0.237*	
H18C	0.1141	0.3350	1.0391	0.237*	
H10	0.342 (4)	0.377 (8)	0.693 (5)	0.07 (2)*	

### Atomic displacement parameters (Å^2^)

**Table:**

	*U*^11^	*U*^22^	*U*^33^	*U*^12^	*U*^13^	*U*^23^
O3	0.066 (2)	0.101 (4)	0.099 (4)	−0.003 (3)	0.016 (3)	−0.005 (3)
N2	0.073 (3)	0.076 (4)	0.056 (3)	−0.017 (3)	0.011 (2)	−0.010 (3)
O5	0.081 (3)	0.119 (5)	0.154 (4)	−0.015 (4)	0.020 (3)	−0.040 (5)
C3	0.059 (3)	0.062 (4)	0.073 (4)	−0.001 (3)	0.015 (3)	−0.002 (3)
O1	0.105 (4)	0.091 (4)	0.092 (4)	0.024 (4)	0.015 (3)	0.000 (4)
C2	0.048 (3)	0.065 (4)	0.057 (3)	−0.007 (3)	0.004 (2)	−0.003 (3)
O6	0.075 (3)	0.172 (7)	0.075 (3)	0.018 (4)	0.004 (2)	−0.009 (4)
O4	0.080 (3)	0.162 (7)	0.097 (4)	−0.018 (4)	0.025 (3)	0.000 (4)
O2	0.090 (3)	0.153 (6)	0.126 (4)	0.051 (4)	0.051 (3)	0.031 (5)
C8	0.072 (4)	0.058 (4)	0.059 (3)	−0.007 (3)	0.003 (3)	0.000 (3)
N1	0.076 (4)	0.093 (5)	0.067 (3)	0.020 (4)	0.011 (3)	−0.001 (4)
C7	0.058 (3)	0.066 (4)	0.051 (3)	−0.007 (3)	0.003 (2)	0.000 (3)
C1	0.053 (3)	0.070 (4)	0.058 (3)	0.005 (3)	0.007 (2)	0.000 (3)
C16	0.068 (4)	0.106 (7)	0.067 (4)	0.001 (5)	0.018 (3)	0.010 (4)
C4	0.076 (4)	0.066 (5)	0.091 (5)	0.002 (4)	0.014 (4)	0.005 (4)
C12	0.090 (5)	0.086 (6)	0.073 (4)	−0.010 (5)	−0.018 (3)	−0.001 (4)
C11	0.062 (3)	0.062 (4)	0.070 (4)	−0.014 (3)	0.006 (3)	−0.002 (3)
O7	0.124 (3)	0.135 (4)	0.131 (3)	−0.003 (3)	0.011 (2)	0.020 (3)
C10	0.071 (4)	0.092 (5)	0.053 (3)	−0.014 (4)	0.005 (3)	0.002 (4)
C6	0.072 (4)	0.084 (5)	0.065 (4)	0.002 (4)	0.019 (3)	0.001 (4)
C13	0.070 (4)	0.127 (7)	0.078 (4)	0.001 (4)	0.010 (3)	−0.023 (3)
C9	0.104 (5)	0.076 (5)	0.060 (4)	−0.021 (4)	0.008 (4)	0.006 (4)
C5	0.090 (5)	0.077 (5)	0.086 (5)	−0.005 (5)	0.018 (4)	0.015 (5)
C17	0.126 (4)	0.131 (4)	0.132 (3)	0.013 (3)	0.031 (3)	−0.018 (3)
C14	0.139 (4)	0.140 (4)	0.130 (3)	0.006 (3)	0.007 (2)	0.017 (3)
C15	0.165 (4)	0.155 (5)	0.134 (3)	0.009 (3)	0.004 (3)	−0.002 (3)
C18	0.168 (5)	0.165 (5)	0.142 (3)	0.009 (3)	0.026 (3)	−0.001 (3)

### Geometric parameters (Å, °)

**Table:**

O3—C11	1.202 (8)	C12—C11	1.501 (9)
N2—C7	1.467 (8)	C12—H12A	0.9600
N2—C10	1.474 (9)	C12—H12B	0.9600
N2—H10	0.92 (5)	C12—H12C	0.9600
O5—C16	1.308 (10)	O7—C13	1.330 (11)
O5—C17	1.470 (11)	O7—C14	1.525 (11)
C3—C4	1.378 (9)	C10—C9	1.518 (10)
C3—C2	1.383 (9)	C10—C13	1.557 (10)
C3—H3	0.9300	C6—C5	1.342 (11)
O1—N1	1.208 (9)	C6—H6	0.9300
C2—C1	1.390 (8)	C9—H9A	0.9700
C2—C7	1.521 (8)	C9—H9B	0.9700
O6—C13	1.162 (9)	C5—H5	0.9300
O4—C16	1.181 (8)	C17—C18	1.509 (9)
O2—N1	1.214 (7)	C17—H17A	0.9700
C8—C11	1.517 (8)	C17—H17B	0.9700
C8—C9	1.537 (8)	C14—C15	1.506 (9)
C8—C7	1.552 (8)	C14—H14A	0.9700
C8—H8	0.9800	C14—H14B	0.9700
N1—C1	1.474 (9)	C15—H15A	0.9600
C7—H7	0.9800	C15—H15B	0.9600
C1—C6	1.382 (9)	C15—H15C	0.9600
C16—C10	1.536 (10)	C18—H18A	0.9600
C4—C5	1.375 (9)	C18—H18B	0.9600
C4—H4	0.9300	C18—H18C	0.9600
			
C7—N2—C10	107.3 (6)	N2—C10—C16	107.8 (6)
C7—N2—H10	105 (4)	C9—C10—C16	114.1 (6)
C10—N2—H10	114 (4)	N2—C10—C13	112.0 (6)
C16—O5—C17	119.2 (7)	C9—C10—C13	112.7 (7)
C4—C3—C2	122.3 (6)	C16—C10—C13	104.9 (5)
C4—C3—H3	118.9	C5—C6—C1	119.7 (6)
C2—C3—H3	118.9	C5—C6—H6	120.1
C3—C2—C1	115.3 (6)	C1—C6—H6	120.1
C3—C2—C7	119.1 (5)	O6—C13—O7	127.1 (9)
C1—C2—C7	125.6 (6)	O6—C13—C10	124.7 (9)
C11—C8—C9	113.0 (5)	O7—C13—C10	108.2 (7)
C11—C8—C7	110.5 (5)	C10—C9—C8	106.3 (6)
C9—C8—C7	103.1 (5)	C10—C9—H9A	110.5
C11—C8—H8	110.0	C8—C9—H9A	110.5
C9—C8—H8	110.0	C10—C9—H9B	110.5
C7—C8—H8	110.0	C8—C9—H9B	110.5
O1—N1—O2	122.1 (8)	H9A—C9—H9B	108.7
O1—N1—C1	119.4 (6)	C6—C5—C4	120.0 (7)
O2—N1—C1	118.5 (8)	C6—C5—H5	120.0
N2—C7—C2	109.7 (6)	C4—C5—H5	120.0
N2—C7—C8	101.8 (5)	O5—C17—C18	101.2 (9)
C2—C7—C8	116.4 (4)	O5—C17—H17A	111.5
N2—C7—H7	109.6	C18—C17—H17A	111.5
C2—C7—H7	109.6	O5—C17—H17B	111.5
C8—C7—H7	109.6	C18—C17—H17B	111.5
C6—C1—C2	122.8 (7)	H17A—C17—H17B	109.3
C6—C1—N1	115.6 (6)	C15—C14—O7	101.4 (8)
C2—C1—N1	121.6 (6)	C15—C14—H14A	111.5
O4—C16—O5	123.5 (8)	O7—C14—H14A	111.5
O4—C16—C10	126.3 (8)	C15—C14—H14B	111.5
O5—C16—C10	110.1 (6)	O7—C14—H14B	111.5
C5—C4—C3	119.8 (7)	H14A—C14—H14B	109.3
C5—C4—H4	120.1	C14—C15—H15A	109.5
C3—C4—H4	120.1	C14—C15—H15B	109.5
C11—C12—H12A	109.5	H15A—C15—H15B	109.5
C11—C12—H12B	109.5	C14—C15—H15C	109.5
H12A—C12—H12B	109.5	H15A—C15—H15C	109.5
C11—C12—H12C	109.5	H15B—C15—H15C	109.5
H12A—C12—H12C	109.5	C17—C18—H18A	109.5
H12B—C12—H12C	109.5	C17—C18—H18B	109.5
O3—C11—C12	121.3 (7)	H18A—C18—H18B	109.5
O3—C11—C8	121.1 (6)	C17—C18—H18C	109.5
C12—C11—C8	117.6 (6)	H18A—C18—H18C	109.5
C13—O7—C14	114.4 (7)	H18B—C18—H18C	109.5
N2—C10—C9	105.4 (5)		
			
C4—C3—C2—C1	0.5 (9)	C7—N2—C10—C16	−150.7 (5)
C4—C3—C2—C7	178.7 (6)	C7—N2—C10—C13	94.5 (7)
C10—N2—C7—C2	164.3 (5)	O4—C16—C10—N2	120.5 (8)
C10—N2—C7—C8	40.5 (6)	O5—C16—C10—N2	−57.8 (8)
C3—C2—C7—N2	−25.6 (7)	O4—C16—C10—C9	3.7 (11)
C1—C2—C7—N2	152.3 (6)	O5—C16—C10—C9	−174.5 (7)
C3—C2—C7—C8	89.2 (7)	O4—C16—C10—C13	−120.0 (9)
C1—C2—C7—C8	−92.9 (7)	O5—C16—C10—C13	61.8 (9)
C11—C8—C7—N2	84.9 (6)	C2—C1—C6—C5	−2.4 (10)
C9—C8—C7—N2	−36.2 (6)	N1—C1—C6—C5	176.5 (7)
C11—C8—C7—C2	−34.3 (8)	C14—O7—C13—O6	−1.7 (14)
C9—C8—C7—C2	−155.3 (6)	C14—O7—C13—C10	178.0 (8)
C3—C2—C1—C6	1.7 (9)	N2—C10—C13—O6	−2.8 (12)
C7—C2—C1—C6	−176.3 (6)	C9—C10—C13—O6	115.9 (10)
C3—C2—C1—N1	−177.2 (6)	C16—C10—C13—O6	−119.5 (10)
C7—C2—C1—N1	4.8 (9)	N2—C10—C13—O7	177.5 (7)
O1—N1—C1—C6	−148.9 (7)	C9—C10—C13—O7	−63.8 (8)
O2—N1—C1—C6	29.6 (9)	C16—C10—C13—O7	60.8 (9)
O1—N1—C1—C2	30.0 (10)	N2—C10—C9—C8	4.1 (8)
O2—N1—C1—C2	−151.4 (6)	C16—C10—C9—C8	122.2 (6)
C17—O5—C16—O4	0.4 (13)	C13—C10—C9—C8	−118.4 (6)
C17—O5—C16—C10	178.7 (7)	C11—C8—C9—C10	−99.6 (7)
C2—C3—C4—C5	−2.0 (11)	C7—C8—C9—C10	19.7 (7)
C9—C8—C11—O3	30.1 (9)	C1—C6—C5—C4	0.8 (11)
C7—C8—C11—O3	−84.8 (8)	C3—C4—C5—C6	1.3 (11)
C9—C8—C11—C12	−150.7 (6)	C16—O5—C17—C18	93.1 (11)
C7—C8—C11—C12	94.4 (7)	C13—O7—C14—C15	119.5 (10)
C7—N2—C10—C9	−28.4 (7)		

### Hydrogen-bond geometry (Å, °)

**Table:**

*D*—H···*A*	*D*—H	H···*A*	*D*···*A*	*D*—H···*A*
N2—H10···O2^i^	0.92 (5)	2.51 (6)	3.244 (9)	138 (5)
C18—H18A···O4^ii^	0.96	2.53	3.460 (15)	162 (6)

Symmetry codes: (i) −*x*+1, *y*+1/2, −*z*+1; (ii) −*x*, *y*+1/2, −*z*+2.
